# Supplementary material for: Common Regulatory Mechanisms Mediated by Cuproptosis Genes in Inflammatory Bowel Disease and Major Depressive Disorder
Source: Genes (Basel). 2025 Mar 14;16(3):339. doi: 10.3390/genes16030339 (PMC11942124; doi:10.3390/genes16030339)
Supplement: Supplementary file 1 [file genes-16-00339-s001.zip › genes-3506739-supplementary.pdf]

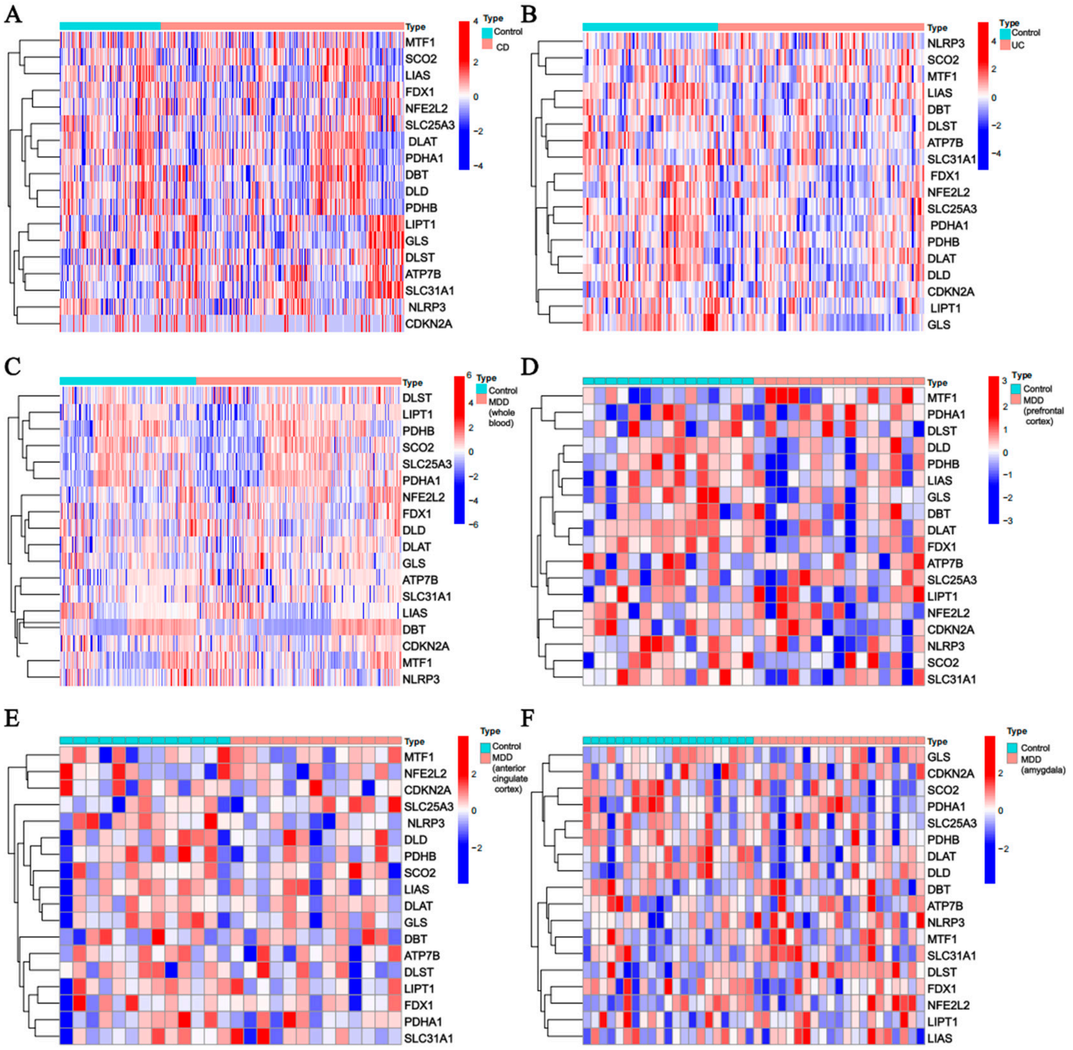

Figure S1 Analysis of 18 CRGs expression patterns in different types of samples using heatmaps. (A) The heat map shows the expression of 18 CRGs in CD colon samples and control samples after merging GSE20881、GSE24287 and GSE179285 databases. (B) The heat map shows the expression of 18 CRGs in UC colon samples and control samples after merging GSE13367、GSE24287 and GSE179285 databases. (C) The heat map shows the expression of 18 CRGs in MDD whole blood samples and control samples after merging GSE98793 and GSE19738 databases. (D) The heat map shows the expression of 18 CRGs in MDD prefrontal cortex samples and control samples in GSE54568 database. (E) The heat map shows the expression of 18 CRGs in MDD anterior cingulate cortex samples and control samples in GSE54571 database. (F) The heat map shows the expression of 18 CRGs in MDD amygdala samples and control samples in GSE54564 database.

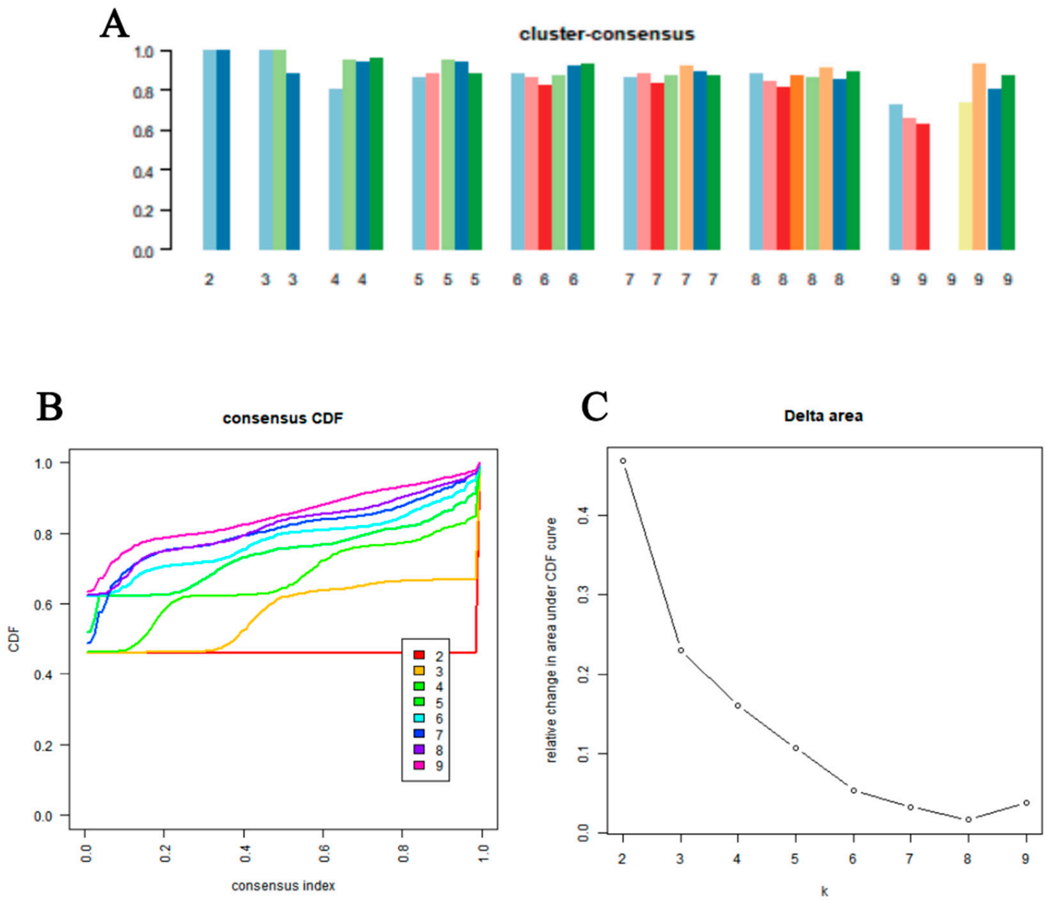

Figure S2 Related indicators of cluster analysis. (A) Scores for consensus clustering. (B) The CDF (cumulative distribution function) curve. (C) The relative change of the area under the CDF curve

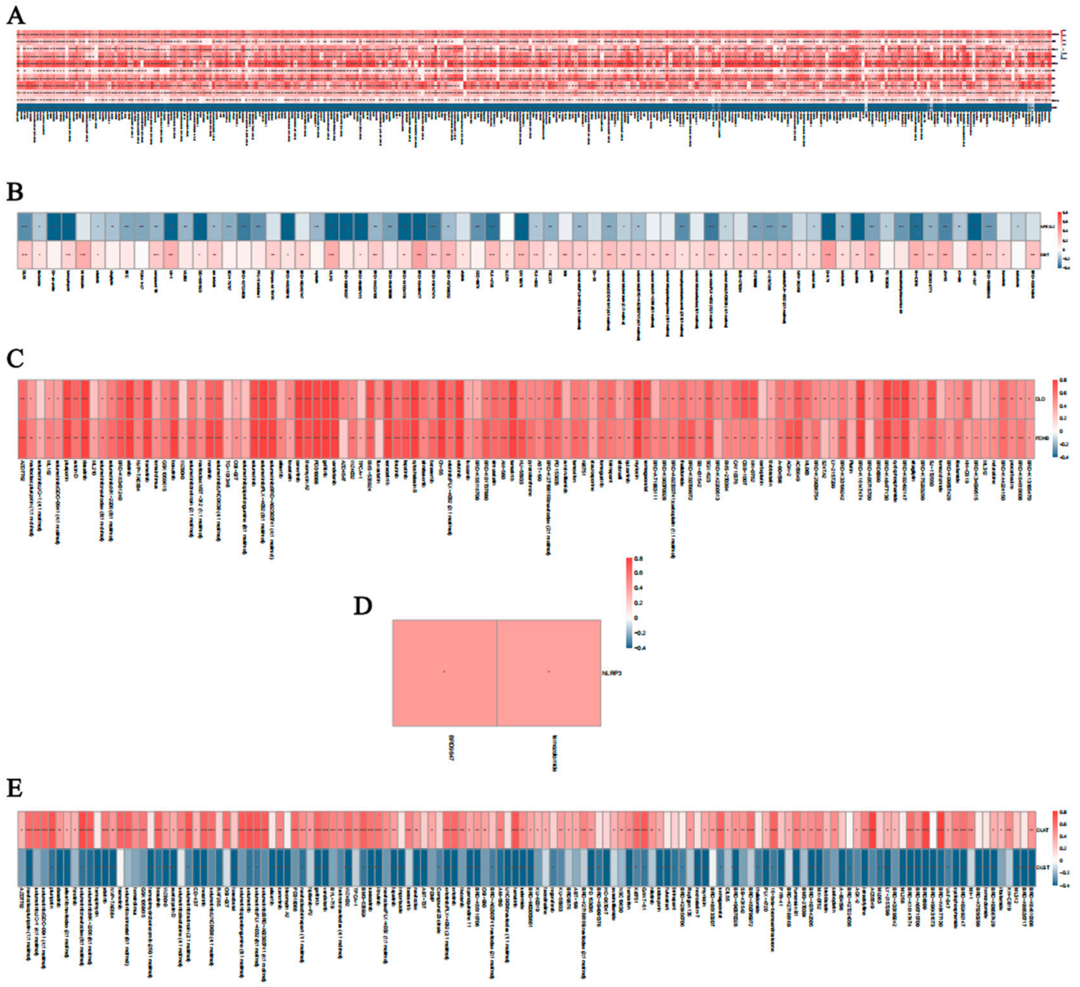

Figure S3 Heatmap analysis of drug sensitivity. Heatmap showing the correlations between drug sensitivity and expression level of DE-CRGs in (A) UC colon samples, (B) MDD whole blood samples, (C) MDD prefrontal cortex samples, (D) MDD anterior cingulate cortex samples and (E) MDD amygdala samples.

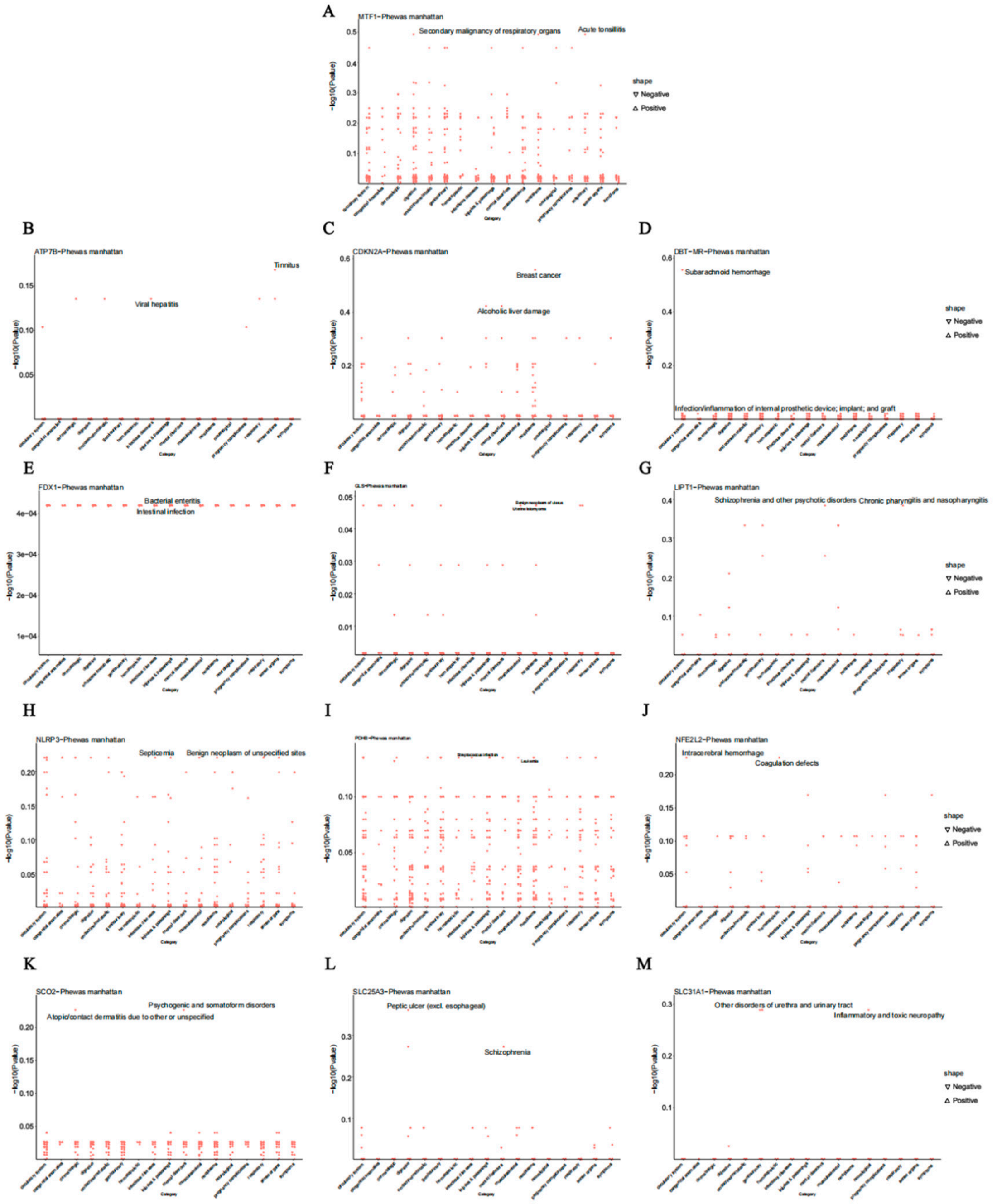

Figure S4 PheWAS analysis of associations between (A) MTF1, (B) ATP7B, (C) CDKN2A, (D) DBT, (E) FDX1, (F) GLS, (G) LIPT1, (H) NLRP3, (I) PDHB, (J) NFE2L2, (K) SCO2, (L) SLC25A3, (M) SLC31A1 and other disease outcomes

Table S1 Potential therapeutic drugs predicted in IBD types and MDD organs.

	IBD	MDD
AC55649	UC	prefrontal cortex , amygdala
afatinib	UC	whole blood , prefrontal cortex , amygdala
AGK-2	UC	prefrontal cortex

**alisertib**	**UC**	**prefrontal cortex， amygdala**
**AM-580**	**UC**	**prefrontal cortex， amygdala**
**avrainvillamide**	**UC**	**amygdala**
**AZD7762**	**UC**	**prefrontal cortex， amygdala**
**barasertib**	**UC**	**prefrontal cortex， amygdala**
**birinapant**	**UC**	**prefrontal cortex， amygdala**
**BMS-270394**	**UC**	**whole blood， prefrontal cortex**
**BMS-536924**	**UC**	**prefrontal cortex， amygdala**
**bosutinib**	**UC**	**prefrontal cortex， amygdala**
**BRD-A05715709**	**CD**	**prefrontal cortex， amygdala**
**BRD-K48477130**	**CD ， UC**	**prefrontal cortex， amygdala**
**BRD-K50799972**	**UC**	**prefrontal cortex**
**BRD-K55116708**	**UC**	**prefrontal cortex， amygdala**
**canertinib**	**UC**	**prefrontal cortex， amygdala**
**carboplatin**	**UC**	**prefrontal cortex， amygdala**
**gefitinib**	**CD**	**whole blood， prefrontal cortex， amygdala**
**GSK1059615**	**UC**	**prefrontal cortex， amygdala**
**Ki8751**	**UC**	**prefrontal cortex， amygdala**
**KU-55933**	**UC**	**prefrontal cortex， amygdala**
**MK-0752**	**CD**	**prefrontal cortex， amygdala**
**neratinib**	**UC**	**prefrontal cortex， amygdala**
**NVP-TAE684**	**UC**	**prefrontal cortex， amygdala**
**OSI-027**	**UC**	**prefrontal cortex， amygdala**
**PD318088**	**UC**	**whole blood， prefrontal cortex， amygdala**
**selumetinib**	**UC**	**whole blood， prefrontal cortex， amygdala**
**temozolomide**	**UC**	**prefrontal cortex， anterior cingulate cortex， amygdala**
**tivozanib**	**UC**	**prefrontal cortex， amygdala**
**TPCA-1**	**UC**	**prefrontal cortex， amygdala**
**WZ8040**	**UC**	**prefrontal cortex， amygdala**
**ELCPK**	**UC**	**whole blood**
**Ch-55**	**UC**	**whole blood， prefrontal cortex**
**tandutinib**	**UC**	**whole blood**
**AT-406**	**UC**	**whole blood**
**WAY-362450**	**UC**	**whole blood**

Table S2 The docking scores between 5 proteins and 37 potential drugs.

	DLD	PDHB	DLAT	DLST	DBT
AC55649	-35.546047	-23.604225	-30.726042	-25.236668	-31.039619
afatinib	-35.130135	-29.25346	-35.084717	-30.311272	-41.020504
AGK-2	-38.10767	-26.413313	-33.208431	-29.736782	-34.336021
alisertib	-36.825089	-28.958641	-42.729588	-35.08271	-36.98201
AM-580	-26.351114	-21.903891	-28.062914	-24.922674	-28.523127
avrainvillamide	-28.646551	-23.760296	-27.782831	-24.515331	-27.244638
AZD7762	-37.264496	-24.073309	-29.905615	-28.712025	-32.067783
barasertib	-43.373829	-33.061508	-46.317345	-40.023647	-42.619648
birinapant	-43.970131	-31.658436	-34.53857	-40.492374	-43.771778
BMS-270394	-33.742626	-25.424089	-28.687687	-27.958063	-31.504293
BMS-536924	-36.496346	-30.543983	-36.241692	-32.81086	-38.192585
bosutinib	-36.629257	-27.380896	-34.63662	-35.210899	-35.323864
BRD-A05715709	-28.231348	-22.351074	-30.564312	-29.501268	-29.246080
BRD-K48477130	-23.118086	-20.987278	-24.110048	-21.243122	-26.822397
BRD-K50799972	-36.273457	-30.661785	-30.557182	-34.596462	-39.763515
BRD-K55116708	-42.028801	-30.169483	-36.45739	-34.813026	-36.885963
canertinib	-36.620682	-30.637857	-38.686234	-34.305462	-38.915230
carboplatin	-14.570821	-13.838824	-15.975746	-14.424491	-16.823040
gefitinib	-35.009331	-26.882109	-34.974609	-31.207918	-36.327900
GSK1059615	-31.373297	-22.901112	-32.845585	-26.304182	-32.631142

**Ki8751**	**-36.229599**	**-25.861052**	**-38.22707**	**-31.3004**	**-34.66687**
**KU-55933**	**-26.277899**	**-19.736252**	**-25.481066**	**-26.938999**	**-30.726528**
**MK-0752**	**-30.697502**	**-24.328329**	**-35.249592**	**-28.227617**	**-24.438675**
**neratinib**	**-44.485619**	**-35.51041**	**-43.602409**	**-37.223713**	**-42.766399**
**NVP-TAE684**	**-37.83387**	**-27.767183**	**-41.444611**	**-33.50391**	**-35.017319**
**OSI-027**	**-32.39613**	**-26.502079**	**-35.998386**	**-29.784145**	**-35.665386**
**PD318088**	**-34.316319**	**-28.198969**	**-36.688404**	**-29.870394**	**-35.713173**
**selumetinib**	**-33.749008**	**-28.633083**	**-34.160282**	**-29.071199**	**-36.299240**
**temozolomide**	**-23.718597**	**-17.942535**	**-20.054916**	**-17.996378**	**-21.739555**
**tivozanib**	**-38.937954**	**-28.935333**	**-34.102886**	**-33.158058**	**-34.911835**
**TPCA-1**	**-29.79512**	**-24.523737**	**-27.791634**	**-23.976372**	**-28.875443**
**WZ8040**	**-41.882748**	**-30.058661**	**-37.528061**	**-31.217936**	**-35.937286**
**ELCPK**	**-34.828026**	**-28.669228**	**-31.216784**	**-28.127089**	**-27.729532**
**Ch-55**	**-33.035851**	**-22.579264**	**-27.768204**	**-26.173271**	**-31.007439**
**tandutinib**	**-34.282944**	**-25.67099**	**-38.981991**	**-33.056824**	**-31.821569**
**AT-406**	**-34.152996**	**-28.396019**	**-25.1506**	**-29.952349**	**-36.094479**
**WAY-362450**	**-28.181072**	**-21.444838**	**-26.542465**	**-28.763241**	**-31.037039**
